# Construction and validation of a novel ferroptosis-related signature for evaluating prognosis and immune microenvironment in ovarian cancer

**DOI:** 10.3389/fgene.2022.1094474

**Published:** 2023-01-05

**Authors:** Jiani Yang, Chao Wang, Shanshan Cheng, Yue Zhang, Yue Jin, Nan Zhang, Yu Wang

**Affiliations:** ^1^ Department of Obstetrics and Gynecology, Shanghai First Maternity and Infant Hospital, School of Medicine, Tongji University, Shanghai, China; ^2^ Shanghai Key Laboratory of Maternal Fetal Medicine, Shanghai First Maternity and Infant Hospital, School of Medicine, Tongji University, Shanghai, China; ^3^ Department of Obstetrics and Gynecology, Renji Hospital, School of Medicine, Shanghai Jiaotong University, Shanghai, China

**Keywords:** ferroptosis, ovarian cancer, gene signature, prognosis, immune microenvironment

## Abstract

Ovarian cancer (OV) is the most lethal form of gynecological malignancy worldwide, with limited therapeutic options and high recurrence rates. However, research focusing on prognostic patterns of ferroptosis-related genes (FRGs) in ovarian cancer is still lacking. From the 6,406 differentially expressed genes (DEGs) between TCGA-OV (*n* = 376) and GTEx cohort (*n* = 180), we identified 63 potential ferroptosis-related genes. Through the LASSO-penalized Cox analysis, 3 prognostic genes, SLC7A11, ZFP36, and TTBK2, were finally distinguished. The time-dependent ROC curves and K-M survival analysis performed powerful prognostic ability of the 3-gene signature. Stepwise, we constructed and validated the nomogram based on the 3-gene signature and clinical features, with promising prognostic value in both TCGA (*p*-value < .0001) and ICGC cohort (*p*-value = .0064). Gene Set Enrichment Analysis elucidated several potential pathways between the groups stratified by 3-gene signature, while the m6A gene analysis implied higher m6A level in the high-risk group. We applied the CIBERSORT algorithm to distinct tumor immune microenvironment between two groups, with less activated dendritic cells (DCs) and plasma cells, more M0 macrophages infiltration, and higher expression of key immune checkpoint molecules (CD274, CTLA4, HAVCR2, and PDCD1LG2) in the high-risk group. In addition, the low-risk group exhibited more favorable immunotherapy and chemotherapy responses. Collectively, our findings provided new prospects in the role of ferroptosis-related genes, as a promising prediction tool for prognosis and immune responses, in order to assist personalized treatment decision-making among ovarian cancer patients.

## 1 Introduction

Ovarian cancer (OV) is the leading cause of death among gynecologic malignant tumors, with 12,810 deaths and 19,880 new cases in the United States, estimated for 2022 (Siegel et al., 2022). Due to the lack of early symptoms or physical signs, over 70% OVs were diagnosed at advanced stage (Ebell et al., 2016). Despite development in therapy over the past decades, the prognosis for patients was still poor, with a 5-year survival rate under 40% (Bray et al., 2018). Moreover, almost 80% of OV patients will suffer cancer recurrence after the initial treatment of standard surgery followed by platinum-based chemotherapy (Jacobs et al., 2016). Given the poor prognosis, identifying potential prognostic signatures and innovative therapeutic targets is urgently needed to improve survival.

Moreover, OV is a highly-heterogeneous carcinoma with a broad spectrum of subtypes, among which the epithelial OV accounts for 50%–70% cases and could be divided into type I, type II and borderline subgroups (Koshiyama et al., 2014). Type I tumors (account for 28% OV cases) mainly includes mucinous (MC), low-grade serous (LGS), endometrioid (END), and clear cell (CCC) carcinomas (Kopper et al., 2019), while Type II tumors (account for 57% OV cases) include high-grade serous ovarian cancer (HGSOC), the driving subtype accounts for 70%–80% of OV mortalities (Cancer Genome Atlas Research, 2011). In this study, we have enrolled in the TCGA-OV cohort as training set, which only consisted of patients with ovarian serous cystadenocarcinoma, the most common serous subtype that represents > 60% of all the cases.

During the past few years, increasing research focused on ferroptosis, a recently recognized form of non-apoptotic programmed cell death, which is driven by the iron-dependent accumulation of lipid reactive oxygen species (ROS) (Mou et al., 2019). Various signaling pathways have been demonstrated to participate in the ferroptosis process, including the MAPK pathway, P53 pathway, and Hippo pathway (Li et al., 2020; Yang et al., 2021b; Chang et al., 2021). With the deepening of research, the importance of ferroptosis has been proved mainly in the regulation of redox biology and metabolism, which could affect the pathogenesis of various cancers, including OV (Liang et al., 2019). Recently, therapeutic approaches targeting ferroptosis-related genes (FRGs) to trigger ferroptosis cell death in OV tissue have attracted considerable attention (Li et al., 2021). Therefore, exploring the underlying functions and mechanisms of FRGs changes in OV is of great significance.

Nowadays, immunotherapy has emerged as a hotspot in the realm of OV treatment (Xu et al., 2021), though the estimated effective rate for cancer immunotherapy is less than 30% (Zhao et al., 2022). Recently, studies indicated that ferroptosis tumor cells in early death stages could induce an adaptive immune response with anti-tumor effects (Efimova et al., 2020). Therefore, ferroptosis activation might become a promising strategy, with great potential to overcome drug resistance to immunotherapy (Xu et al., 2021). However, to date, the importance of FRGs in OV prognosis and immunotherapy has been rarely reported.

In the present study, we comprehensively analyzed the vital role of FRGs in OV and developed a promising prognostic model based on the FRGs selected. Additionally, we also evaluated the tumor immune microenvironment, methylation of N6 adenosine (m6A) level, and immunotherapy/chemotherapy response between groups stratified by the FRGs-based signature.

## 2 Materials and methods

### 2.1 Data collection

**FIGURE 1:**
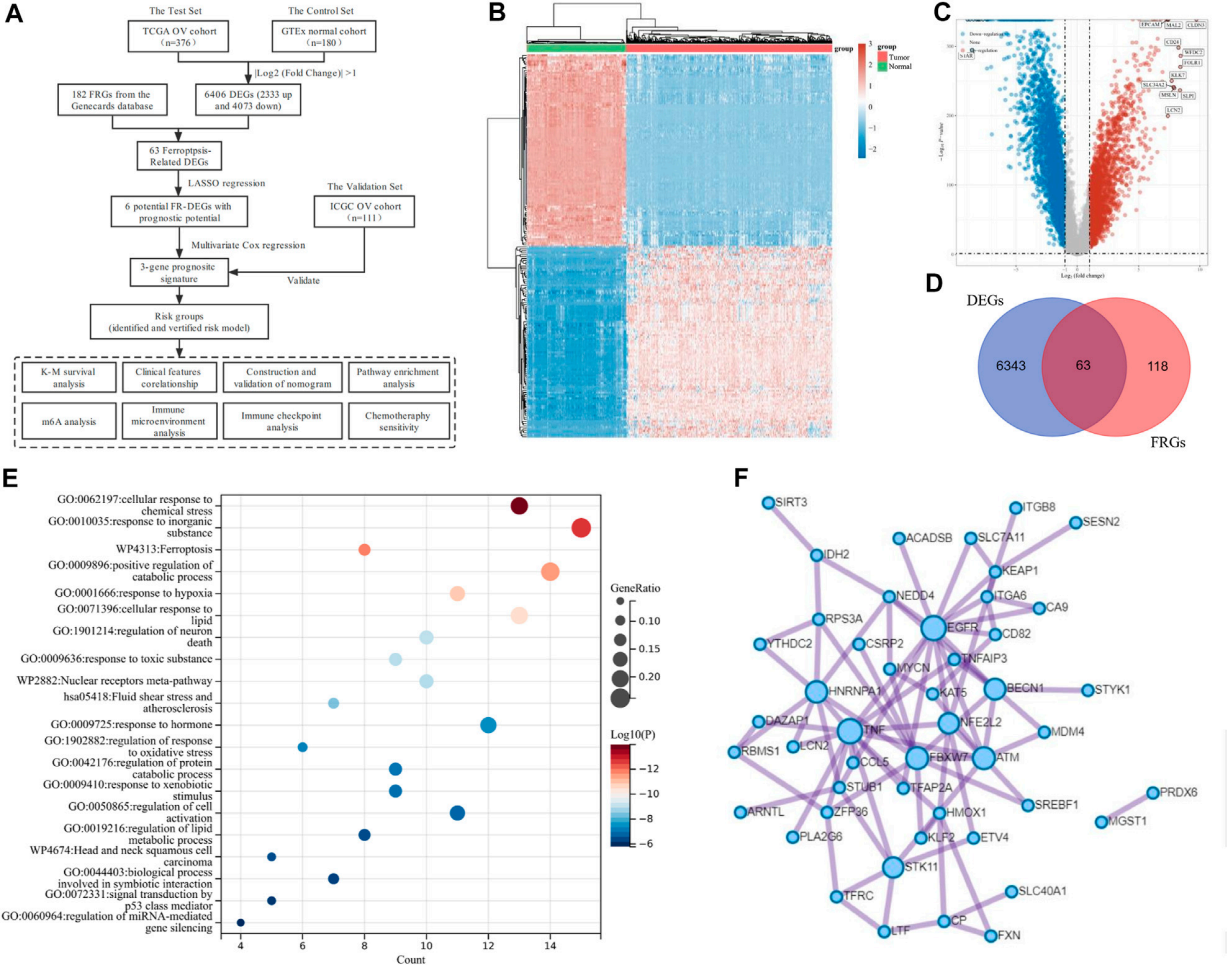
Identification of Ferroptosis-Related Differentially Expressed genes (DEGs) in ovarian cancer (OV). **(A)** The overall flowchart of the study. **(B)** The heatmap for differential gene expression. Different colors represent the trend of gene expression between OV and normal tissues. The top 50 downregulated and top 50 upregulated genes were shown. **(C)** The volcano plot was constructed to list the DEGs between OV and normal tissues *via* the fold change values and P-adjust. The downregulated and upregulated DEGs were highlighted in blue and red, respectively. **(D)** The Venn diagram of the differentially expressed ferroptosis-related genes (DE-FRGs). **(E)** Overview of the Kyoto Encyclopedia of Genes and Genomes (KEGG) and Gene Ontology (GO) pathway enrichment analysis of primary biological actions for the 63 DE-FRGs. Here, the top 20 clusters were listed. The color scale represented *p*-value, and the size of the circles represented the gene ratio. **(F)** The protein-protein interaction (PPI) network of the DE-FRGs.

The RNA-sequencing expression profiles (level 3) and corresponding clinical information of 376 OV tumor samples were downloaded from the TCGA database (https://portal.gdc.com) up to April 2022. Meanwhile, the transcriptome data from 180 normal tissues were downloaded as controls from the GTEx database (https://gtexportal.org/home/datasets), as depicted in Figure 1A. We normalized the gene expression profiles *via* the limma package in the R software. Based on the set cut-off criteria of |Log2 (Fold Change)| >1 and adjusted *p*-value < .05, the differentially-expressed genes (DEGs) between OV and normal tissues were identified. Additionally, the RNA-sequencing expression profiles and corresponding clinical information of 111 OV patients were downloaded from the ICGC database (https://dcc.icgc.org/releases/current/Projects) as the validation set. The workflow of the study is shown in Figure 1A.

### 2.2 Selection of ferroptosis-related genes

To identify ferroptosis-related mRNAs of OV patients, we downloaded a total of 182 FRGs (Relevance score ≥ 1) from the Genecards database (https://www.genecards.org/). Through the Venn diagram (http://bioinformatics.psb.ugent.be/webtools/Venn/), we selected FRGs, which are differentially expressed between OV tumor tissues and normal tissues. The multi-gene correlation heatmap was displayed through the “ggstatsplot” package in the R software package. For Kaplan-Meier (K-M) curves of the genes, the *p*-value and hazard ratio (HR) with 95% confidence interval (95% CI) were generated *via* Log-rank tests.

### 2.3 Construction and validation of the prognostic signature

The least absolute shrinkage and selection operator (LASSO) regression algorithm with 10-fold cross-validation was performed for the feature selection of FRGs with prognostic value. Multivariate Cox regression analyses were used to build the FRGs-based prognostic model. The “glmnet” R package was used to identify prognostic gene signatures and calculate the risk score of each patient in the datasets based on the signature. For survival analysis, the samples were divided into low-risk and high-risk groups based on the medium cut-off value, and the K-M analysis was used to explore the prognostic significance of the signature. Time-dependent receiver operating characteristic curve (ROC) analysis of 1-year, 3-year, and 5-year survival was analyzed using the “timeROC” R package.

Next, both univariate and multivariate Cox regression analyses were performed to assess the independent prognostic factors to build the nomogram. The forestplot was used to show the *p*-value and HR (95% CI) of each parameter, using the “forestplot” R package. Based on the selected prognostic parameters, a prognostic nomogram was developed to predict the 1-year, 3-year, and 5-year OS of OV patients in the TCGA dataset, using the “rms” R package. We measured the discrimination of the nomogram model through calibration curves, which could overlay the observed probabilities and nomogram-predicted probabilities with 95% CI. Moreover, we validated the model *via* the Harrell’s concordance index (C-index) with a 10-fold cross-validation.

### 2.4 RNA isolation and quantitative real-time PCR

OV tissues were obtained from 36 OV patients, who have received cytoreductive surgery, followed by platinum-based chemotherapy, at the Department of Gynecology in Renji Hospital from 5 Marc 2019 to 16 December 2019. OS (overall survival) was measured from the date of initial surgery to the last follow-up or death. Progression-free survival (PFS) was identified from initial treatment to the last follow-up or OV progression, which was assessed by clinical and radiographic evidence. This project was approved by the Ethics Committee of Renji Hospital Affiliated to Shanghai Jiaotong University School of Medicine. All patients provided informed consents, in compliance with the declaration of Helsinki, for the usage of their samples for research purposes.

Total RNA of the tissues was extracted by the Trizol Reagent (T9424, Merk) and then reverse transcribed into cDNA by the RevertAid First Strand Cdna Synthesis Kit (K1622, Thermo Fisher Scientific) following the protocols. Then, we conducted the real-time quantitative reverse transcription-polymerase chain reaction (RT-PCR) analysis through the SYBR Green Master Mix (A25742, Thermo Fisher Scientific) using the QuantStudio™ 7 Flex Real-Time PCR System (Life technologies, United States) following manufacturer’s instructions. Primer sequences were designed as follows: SLC7A11, Forward: 5′—TCA​TTG​GAG​CAG​GAA​TCT​TCA—3′ and Reverse: 5′—TTC​AGC​ATA​AGA​CAA​AGC​TCC​A—3′; ZFP36, Forward: 5′—CCC​AAA​TAC​AAG​ACG​GAA​CT—3′ and Reverse: 5′—GCT​CTG​GCG​AAG​CAC​A—3′; TTBK2, Forward: 5′—ATG​CTC​ACC​AGG​GAG​AAT​GT—3′ and Reverse: 5′—TGC​ATG​ACC​ACG​TAG​TTG​AAA—3′; and GAPDH, Forward: 5′—GGC​AAA​TTC​CAT​GGC​ACC​G—3′ and Reverse: 5′—TCG​CCC​CAC​TTG​ATT​TTG​GA—3′. The comparative expression level was calculated by the 2^−ΔΔCT^ method. GAPDH was used as an internal control.

### 2.5 Functional enrichment analysis

To confirm underlying functions and associated high-level genome information of potential genes, we analyzed the data *via* functional enrichment. We conducted Gene Ontology (GO, including molecular function, biological pathways, and cellular components) and Kyoto Encyclopedia of Genes and Genomes (KEGG) enrichment analyses by Metascape (https://metascape.org), a public-available online tool to analyze DEGs from multiple data sets (Zhou et al., 2019). Stepwise, we processed the DEGs through the Search Tool for the Retrieval of Interacting Genes (STRING, https://string-db.org), a website that could provide screens for human–protein interactions (von Mering et al., 2003), after which the Cytoscape software was used to generate a visualized protein-protein interaction (PPI) network.

### 2.6 Immune infiltrate analysis

To characterize the tumor immune microenvironment, we estimated the abundance of tumor-infiltrating immune cells (TIICs) of each sample through the CIBERSORT algorithm (Newman et al., 2015), a immunological computing method based on a gene expression signature matrix with various marker genes. Original gene expression data from TCGA was normalized before CIBERSORT analysis. Then, at the CIBERSORTx website (https://cibersortx.stanford.edu/), we downloaded the gene signature matrix with interpretation, which outlined 22 subtypes of TIICs. In order to enhance deconvolution algorithm’s accuracy, we accounted for the *p*-value and root mean squared error of the CIBERSORT. The correlation between the risk score of the signature and immune cells was calculated *via* the Spearman’s correlation test.

### 2.7 Impact of risk scoring system on ovarian cancer patients receiving immunotherapy and chemotherapy

The Pearson’s test was performed to assess the association between the signature and expression of immune checkpoint genes, such as cytotoxic T-lymphocyte-associated protein 4 (CTLA4), programmed cell death protein 1 (PDCD1), and programmed cell death protein 1 ligand 2 (PDCD1LG2), etc. Additionally, based on the RNA-sequencing expression profiles and corresponding clinical data from the TCGA database, we evaluated the potential Immune Checkpoint Blockade (ICB) response. Stepwise, we predicted the potential ICB response of OV patients through the Tumor Immune Dysfunction and Exclusion (TIDE) algorithm (http://tide.dfci.harvard.edu), a public-available computational framework which was developed by Jiang et al. (2018) to model tumor immune escape and predict ICB response.

Furthermore, we predict the chemotherapeutic response of each patient based on the Genomics of Drug Sensitivity in Cancer (GDSC, https://www.cancerrxgene.org/), the largest public-available pharmacogenomics database. The prediction process was conducted using the “pRRophetic” R package. To identify effective drugs for OV treatment, the samples’ half-maximal inhibitory concentration values (IC50) were obtained from the GDSC database and estimated by ridge regression.

### 2.8 Statistical analysis

3 All the bioinformatic statistical analyses were implemented by the R software (foundation for statistical computing 2020, version 4.0.3). All the *p*-values had been passed a two-tailed test, and *p*-value < .05 was considered statistically significant. Differences between the low-risk and high-risk groups were compared using the Wilcoxon test, and *p*-values were adjusted through the BH method. Spearman correlation analysis was applied to estimate the correlation between quantitative variables without normal distribution.

## 3 Results

### 3.1 Identification of ferroptosis-related differentially expressed genes in OV

The transcriptome data and corresponding clinical characteristics of 376 OV patients were obtained from the TCGA database. Meanwhile, the transcriptome data from 180 normal tissues were also downloaded as controls from the GTEx database. A total of 6,406 DEGs were identified, among which 2,333 genes were upregulated, and 4,073 genes were downregulated in OV, compared to the normal controls (Figures 1B,C). Then, 182 FRGs (Relevance score ≥ 1) were downloaded from the Genecards database for our further analyses, among which 63 FRGs were differentially expressed between tumor tissues and normal tissues, as shown in the Venn diagram (http://bioinformatics.psb.ugent.be/webtools/Venn/) (Figure 1D). The enrichment analysis of the 63 differentially expressed ferroptosis-related genes (DE-FRGs) was processed by Metascape (https://metascape.org) (Zhou et al., 2019), which exported the top 20 most significant KEGG and GO pathways in the TCGA-OV cohort (Figure 1E). The pathways were mainly enriched in cellular response to chemical stress, response to inorganic substances, ferroptosis, etc. In order to obtain a PPI network, the DE-FRGs were processed through the Search Tool for the Retrieval of Interacting Genes (STRING, https://string-db.org), a website that could provide screens for human–protein interactions (von Mering et al., 2003) (Figure 1F).

### 3.2 Establishment and estimation of prognostic signature based on ferroptosis-related genes

**FIGURE 2:**
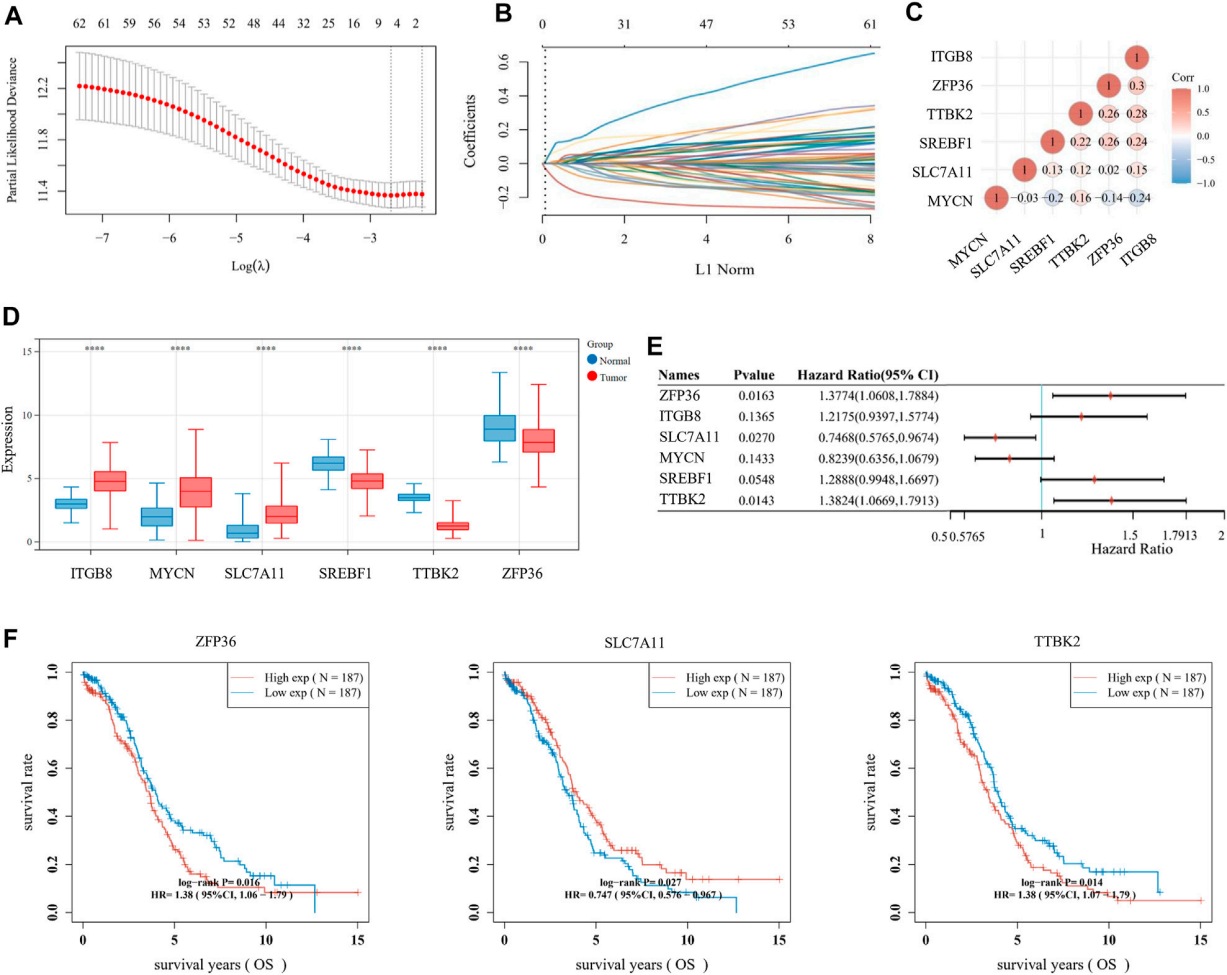
Establishment of prognostic signature based on ferroptosis-related genes (FRGs). **(A)** The *λ* selection diagram for 10-fold cross-validation of tuning parameter selection in the LASSO model. **(B)** The LASSO Cox analysis of the optimal prognostic FRGs, including ZFP36, ITGB8, SLC7A11, MYCN, SREBF1, and TTBK2. The coefficients of the selected features are shown *via* the lambda parameter. **(C)** The expression distribution of the six optimal prognostic FRGs in ovarian cancer (OV) tumor tissues and normal tissues. **(D)** The heatmap of correlation among the six optimal prognostic FRGs. The ordinate and abscissa represent genes, while colors represent different correlation coefficients (blue for positive correlation and red for negative correlation). **(E)** The forest plot of the prognostic ability of the six optimal FRLs, analyzed *via* the multivariate Cox hazard regression method. **(F)** The K-M survival curves of three prognostic FRLs, namely SLC7A11, ZFP36, and TTBK2.

For multiple regression analysis, the LASSO-penalized Cox analysis is a common method to enhance model explicability and forecast accuracy. From the above-mentioned 63 DE-FRGs, six genes (ZFP36, ITGB8, SLC7A11, MYCN, SREBF1, and TTBK2) were filtered to be related to OV patient prognosis through the LASSO regression model (Figures 2A, B). The overview of the function of the six potential DE-FRGs was listed in Table 1 (Helland et al., 2011; Nie et al., 2013; Liao et al., 2015; Cui et al., 2018; Moore et al., 2018; Zhu et al., 2020; Hong et al., 2021). The expression distribution of the six optimal prognostic FRGs in OV tumor tissues and normal tissues was shown in Figure 2C, and the correlation among the FRGs was also presented (Figure 2D). Next, we used the multivariate Cox hazard regression analysis to distinguish prognostic genes, namely SLC7A11, ZFP36, and TTBK2 (Figure 2E). Therefore, the three-gene prognostic signature model was ultimately constructed as follows: Risk score = (−.2084) * SLC7A11+ (.0945) * ZFP36 + (.3619) * TTBK2. The K-M survival curves showed that OV patients with high expression of ZFP36 and TTBK2 suffered worse OS, while those with high expression of SLC7A11 had better OS (Figure 2F).

**TABLE 1:** Overview of the six differentially expressed ferroptosis-related genes (DE-FRGs) with prognosis value in ovarian cancer (OV) (Cui et al., 2018, Helland et al., 2011, Hong et al., 2021, Liao et al., 2015, Moore et al., 2018, Nie et al., 2013, Zhu et al., 2020).

Gene symbol	Gene name	Function in OV	References
ITGB8	Integrin subunit beta 8	Ectopic expression of ITGB8 alleviated the cisplatin sensitivity through the regulation of the cell cycle (decreased G1 phase but an increased S phase) and motility in OV.	Cui et al. (2018), Zhu et al. (2020)
MYCN	BHLH transcription factor	Amplification of MYCN influences the regulatory loop involving HMGA2, Let-7, and LIN28B, which is related to a specific OV subtype with sparse immune cell infiltration.	Helland et al. (2011)
SLC7A11	Solute carrier family 7 member 11	SLC7A11 could influence the ferroptosis process in OV by mediating the cysteine uptake and promoting GSH biosynthesis.	Hong et al. (2021)
SREBF1	Sterol regulatory element-binding transcription factor 1	SREBP1 regulates OV cell growth and survival *via* accommodating downstream lipogenic genes.	Nie et al. (2013)
TTBK2	Tau tubulin kinase 2	Unknow in OV. TTBK2, as a serine/threonine-protein kinase in the CK1 superfamily, could phosphorylate tau and tubulin, which has been implicated in tumor progression.	Liao et al. (2015)
ZFP36	Zinc finger protein 36	Unknow in OV. ZFP36, as an RNA-binding protein, is a prominent inflammatory regulator linked to autoimmunity and cancer.	Moore et al. (2018)

**FIGURE 3:**
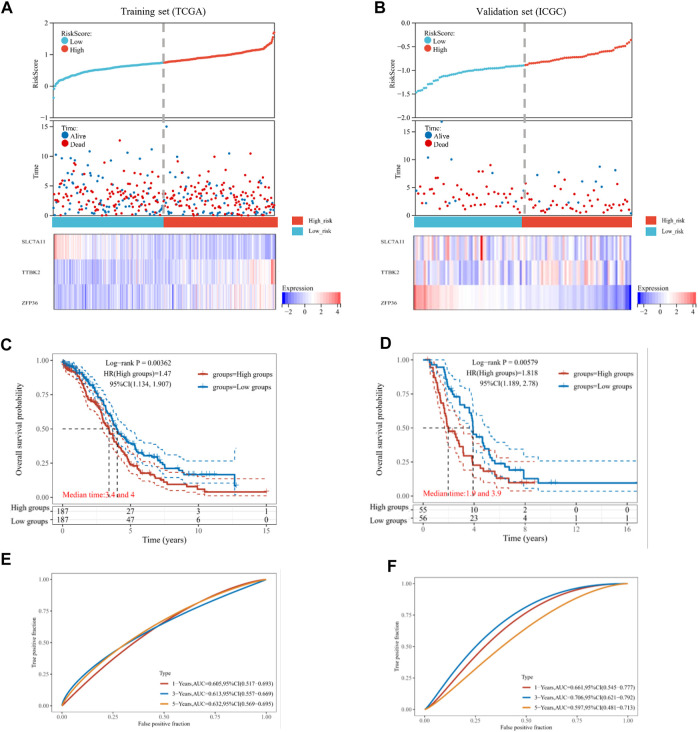
Estimation of prognostic signature based on ferroptosis-related genes (FRGs). The distribution of risk score, survival time, and survival status for each ovarian cancer (OV) patient in the training set **(A)** and validation set **(B)**. The scatter plot distribution represented the risk score of different samples corresponding to the survival time and status (top and middle). The heatmap is the gene expression of the three-gene signature (bottom). The Kaplan-Meier (K-M) curves for patient overall survival (OS) in the high-risk group and low-risk group of the training set **(C)** and validation set **(D)**. The ROC analysis of the training set **(E)** and validation set **(F)** for OS prediction by the three-gene signature.

We focused on the TCGA database as the training set (*n* = 374) and the ICGC database as the validation set (*n* = 111). Then, the risk score of every OV patient was calculated *via* the formula mentioned above. Based on the median cut-off point, we divided OV patients into two groups: high-risk and low-risk in both training and validation sets (Figures 3A, B, top). We also showed the survival status of all OV patients and the expression profiles of the three prognostic genes in high-risk and low-risk groups (Figures 3A, B, middle and bottom). Most of the death cases were mainly distributed in the high-risk group. Additionally, TTBK2 was highly expressed in the high-risk group, while SLC7A11 was highly expressed in the low-risk group. The K-M survival curves showed that the high-risk group suffered worse 1-year, 3-year, and 5-year OS, compared with the low-risk group in the training set (*p*-value = .00362) and validation set (*p*-value = .00579) (Figures 3C, D). Additionally, the ferroptosis-associated three-gene prognostic signature showed promising AUC values in the time-dependent ROC analysis for 1-year, 3-year, and 5-year OS ((Figures 3E, F). Taken together, the ferroptosis-associated three genes were prognostic signature for OV patients.

### 3.3 Construction and validation of the ferroptosis-associated three-gene-based nomogram

**FIGURE 4:**
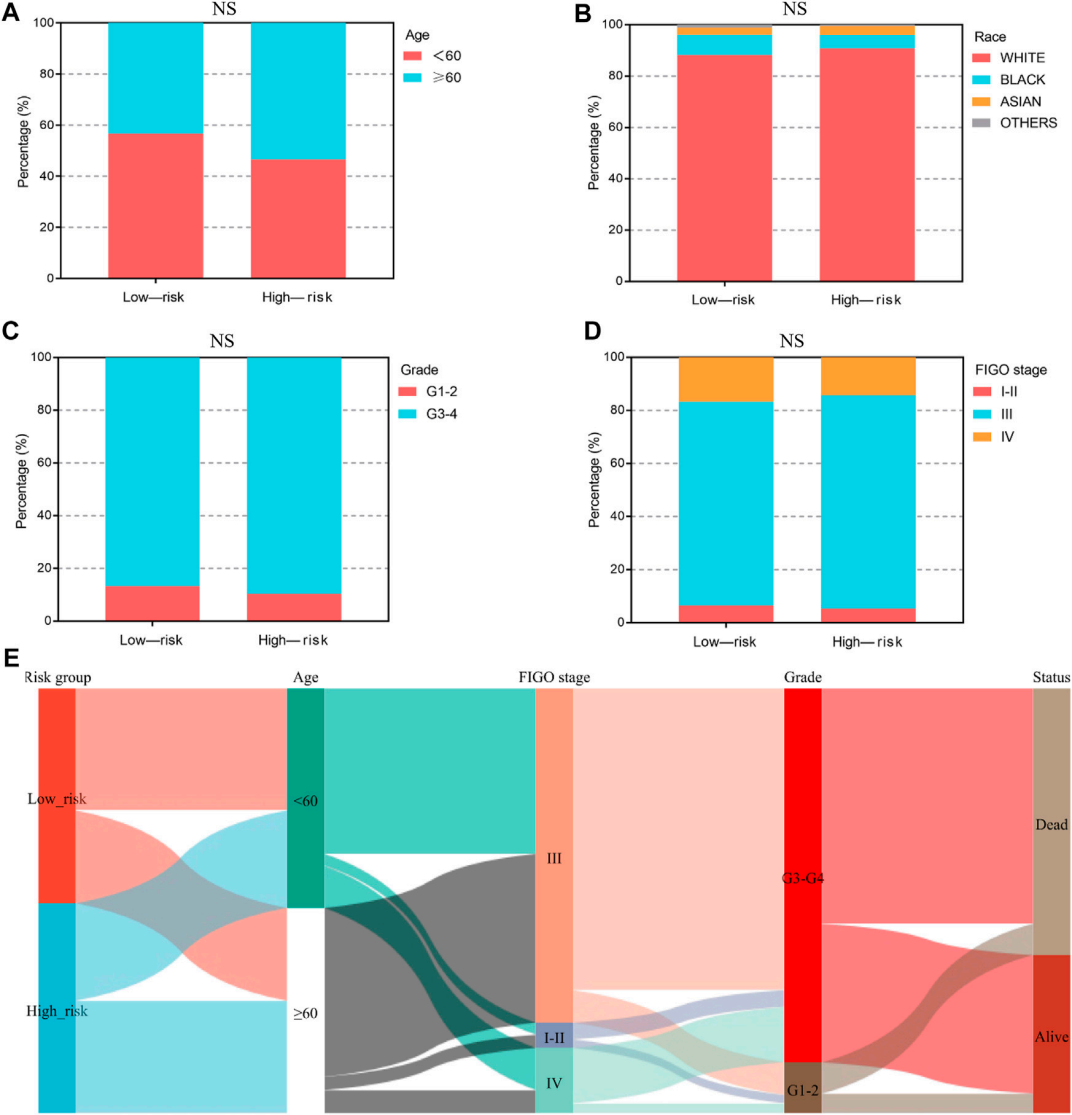
The clinical characteristics of OV patients, stratified by the ferroptosis-associated 3-gene signature. **(A–D)** The stacked bar chart for clinical features distribution, including age, race, grade, and FIGO stage, among the low-risk and high-risk groups. The significance *p*-value was analyzed *via* the chi-square test. NS, no significance. **(E)** The Sankey diagram for features, including age, grade, FIGO stage, and the 3-gene signature. Each color represents different typing, each row represents a different variable, and each line represents the distribution of the same sample refer to different variables.

**FIGURE 5:**
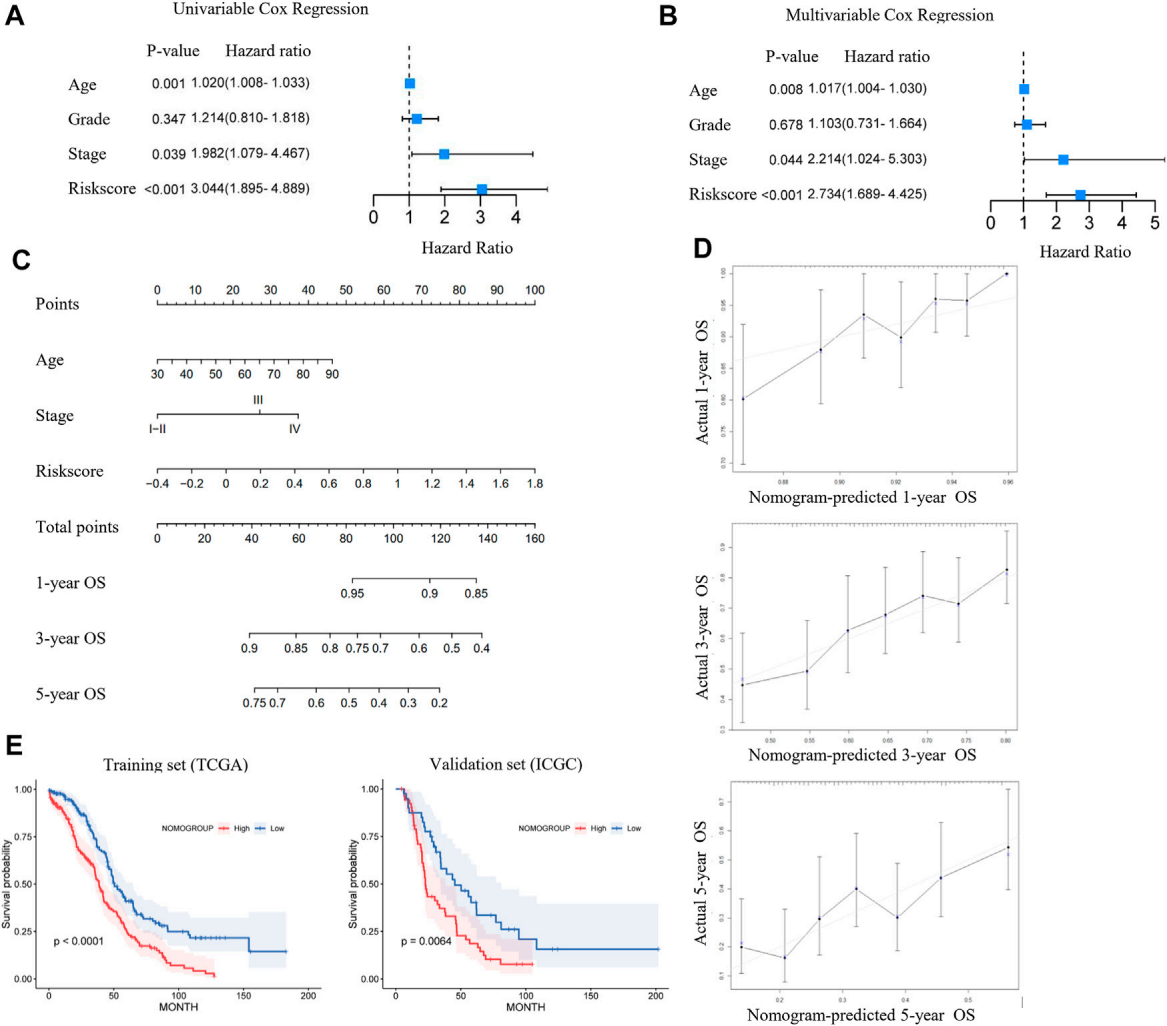
Construction and validation of the ferroptosis-associated three-gene-based nomogram. The forest plot of univariate **(A)** and multivariate **(B)** Cox regression analysis for ovarian cancer (OV) patient survival, based on the 3-gene signature and clinical features. **(C)** The Nomogram model of the risk score and clinical indicators for predicting 1-year, 3-year, and 5-year OS of OV patients in the TCGA cohort. **(D)** The calibration plots of the nomogram for predicting 1-year (top), 3-year (middle), and 5-year (bottom) OS of OV patients. The dotted line indicated the actual survival. **(E)** The Kaplan-Meier (K-M) curves for OV patients in the training (TCGA) and validation cohort (ICGC), stratified by the nomogram score.

We analyzed the relationship between the ferroptosis-associated 3-gene signature and clinical characteristics (Figures 4A–D). The results indicated that features including age, race, grade, and FIGO stage had no significance with the signature (*p*-value ≥ .05). Figure 4E showed the distribution of each OV patient, referring to different clinical variables and the risk groups stratified by the 3-gene signature. In addition, we conducted both univariate and multivariate Cox regression analyses to find out whether risk score was an independent prognostic factor for OV patients (Figures 5A, B). Through the multivariate Cox regression analyses, we found that in addition to risk score (*p*-value < .001), FIGO clinical stage (*p*-value = .044) and age (*p*-value = .008) were also confirmed as prognostic factors. Based on the integration of risk score, FIGO clinical stage, and age, we constructed a nomogram of 1-year, 3-year, and 5-year OS probability among OV patients, with the concordance index (C-index) of .6334 (Figure 5C). Calibration curves of the nomogram implied excellent consistency with standard curves between observed and predicted 1-year, 3-year, and 5-year outcomes (Figure 5D). Furthermore, we calculated the nomogram score of every OV patient and divided them into two groups based on the median cut-off point. The K-M survival curves showed that OV patients with high nomogram scores suffered worse OS in both training and validation sets (Figure 5E). Thus, our results indicated that the nomogram model based on the 3 FRGs could predict OV patient prognosis efficiently.

### 3.4 The ferroptosis-related genes predict prognosis in OV patients

**FIGURE 6:**
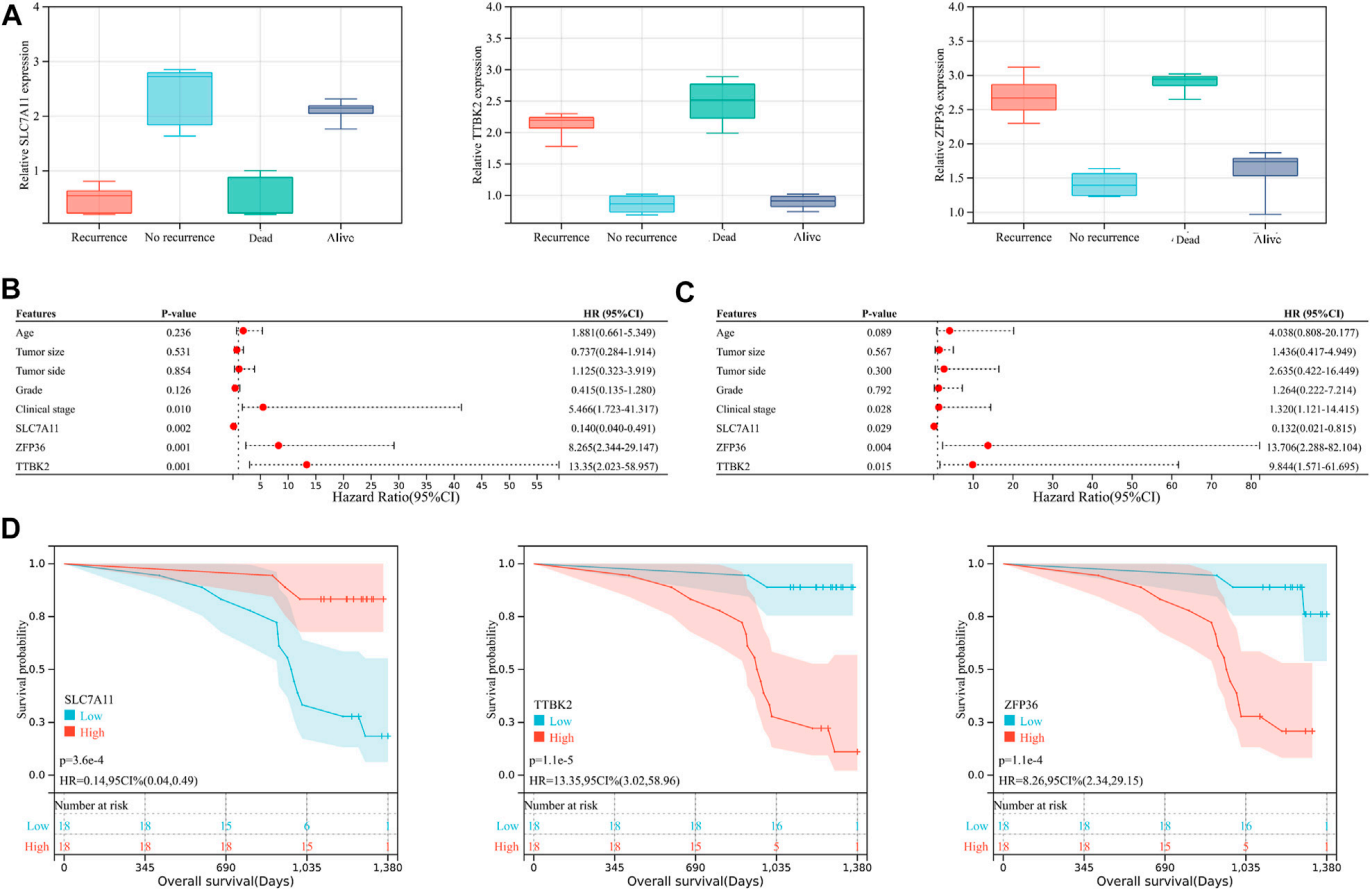
The ferroptosis-related genes predict prognosis in ovarian cancer (OV) patients. **(A)** The expression of SLC7A11, ZFP36, and TTBK2 in the OV tissues, which was measured by qRT-PCR analysis. The forest plot of univariate **(B)** and multivariate **(C)** Cox regression analysis for OV patient survival, based on the 3-gene signature and clinical features. **(D)** The Kaplan-Meier (K-M) curves for OV patients, stratified by the expression of SLC7A11, ZFP36, and TTBK2.

However, considering that the findings of bioinformatics analysis based on public database have many uncertainties, we tried to verify the correctness through experiments on human tissue. We enrolled in 36 OV patients at our institution, with the medium follow-up time of 37.88 (31.48–42.72) months. The clinical features of the involved patients were listed in Table 2. We conducted the qRT-PCR analysis to measure the expression of SLC7A11, ZFP36, and TTBK2 in the OV tissues. The results revealed that higher expression of ZFP36 and TTBK2 was found in OV patients who suffered poor prognosis, while lower SLC7A11 expression were found among them (*p*-value < .05, Figure 6A).

**TABLE 2:** The baseline information of ovarian cancer (OV) patients.

Characteristics	Number of cases (n, %)
Age	
<60 years	15 (41.67%)
≥60 years	21 (58.33%)
Tumor size	
<6 cm	17 (47.22%)
≥6 cm	19 (52.78%)
FIGO stage	
I–II	8 (22.22%)
III–IV	28 (77.78%)
Pathological grade	
G1–2	5 (13.89%)
G3	31 (86.11%)
Ascites	
<1,000 ml	18 (50.00%)
≥1,000 ml	18 (50.00%)
Survival status	
Alive	19 (52.78%)
Dead	17 (47.22%)

Stepwise, to better evaluate the significance of ferroptosis-related genes among OV patients, we performed both univariate and multivariate Cox regression analyses for clinical features in relation to OV patient prognosis (Figures 6B,C). The results indicated that SLC7A11, ZFP36, and TTBK2 (*p*-value = .029, .004, and .015, respectively) were prognostic factors, in addition to FIGO clinical stage (*p*-value = .028). The K-M survival curves showed that patients with higher expression of ZFP36 and TTBK2 suffered worse OS, while those with high expression of SLC7A11 had better prognosis (*p*-value < .05, Figure 6D), which was consist with the results of bioinformatics analysis.

### 3.5 Defining FRGs-related pathways by gene set enrichment analysis

**FIGURE 7:**
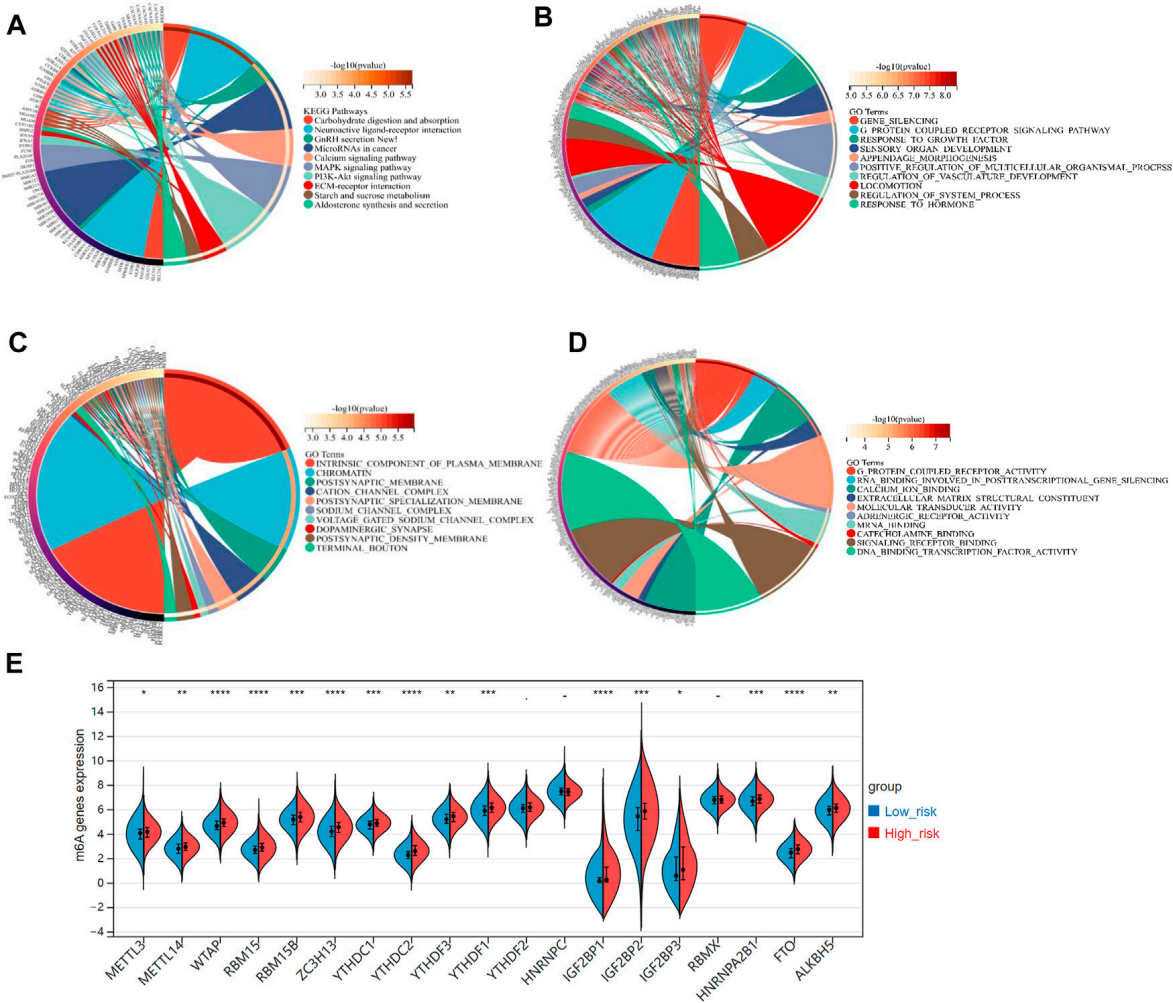
**(A)** The KEGG analysis of primary biological actions for potential mRNA. **(B–D)** The GO analysis of potential targets of mRNAs was also conducted in the aspects of biological process (BP), cellular component (CC), and molecular function (MF). The color scale represented *p*-value, and the size of the circles represented gene numbers. **(E)** The violin plot for the expression distribution of 19 m6A-related genes in low-risk and high-risk groups. *p-value < .05; **p-value < .01; ***p-value < .001; ****p-value < .0001.

Stepwise, we defined the FRGs-related pathways through enrichment analysis among the groups stratified by the 3-gene signature. The KEGG pathway enrichment analysis identified several critical pathways, such as the microRNAs in cancer, PI3K-Akt signaling pathway, MAPK signaling pathway, and others (Figure 7A). As shown in Figure 7B, interestingly, the GO biological process (BP) pathways were mainly enriched in those related to gene silencing, G-protein coupled receptor signaling pathway, response to growth factors, etc. The GO cellular component (CC) pathway analysis identified significantly enriched pathways, including chromatin, terminal bouton, intrinsic component of the plasma membrane, and others (Figure 7C). In Figure 7D, the GO molecular function (MF) pathways were mainly enriched in RNA binding involved in post-transcriptional gene silencing, mRNA binding, DNA binding transcription factor activity, etc. Collectively, pathways related to gene silence and RNA modification were significantly enriched.

Recently, researchers reported that the m6A, a common type of RNA modification, played a critical role in cancer development and progression (Shi et al., 2019). Accordingly, we derived 19 typical m6A-related genes (including METTL3, METTL14, RBM15B, RBM15, MTAP, YTHDC1, YTHDC2, ZC3H13, YTHDF1, YTHDF2, YTHDF3, IGF2BP1, IGF2BP2, IGF2BP3, HNRNPC, HNRNPA2B1, RBMX, ALKBH5, and FTO) from a study on the clinical significance and molecular characterization of m6A modulators of 33 cancer types in the TCGA pan-cancer project (Li et al., 2019). Interestingly, all these m6A-related genes, expected for YTHDC2, HNRNPC, and RBMX, were significant-highly expressed in the high-risk group, compared to the low-risk group (*p*-value < .05, Figure 7E).

### 3.6 Immunity analysis for the risk score and tumor immune microenvironment

**FIGURE 8:**
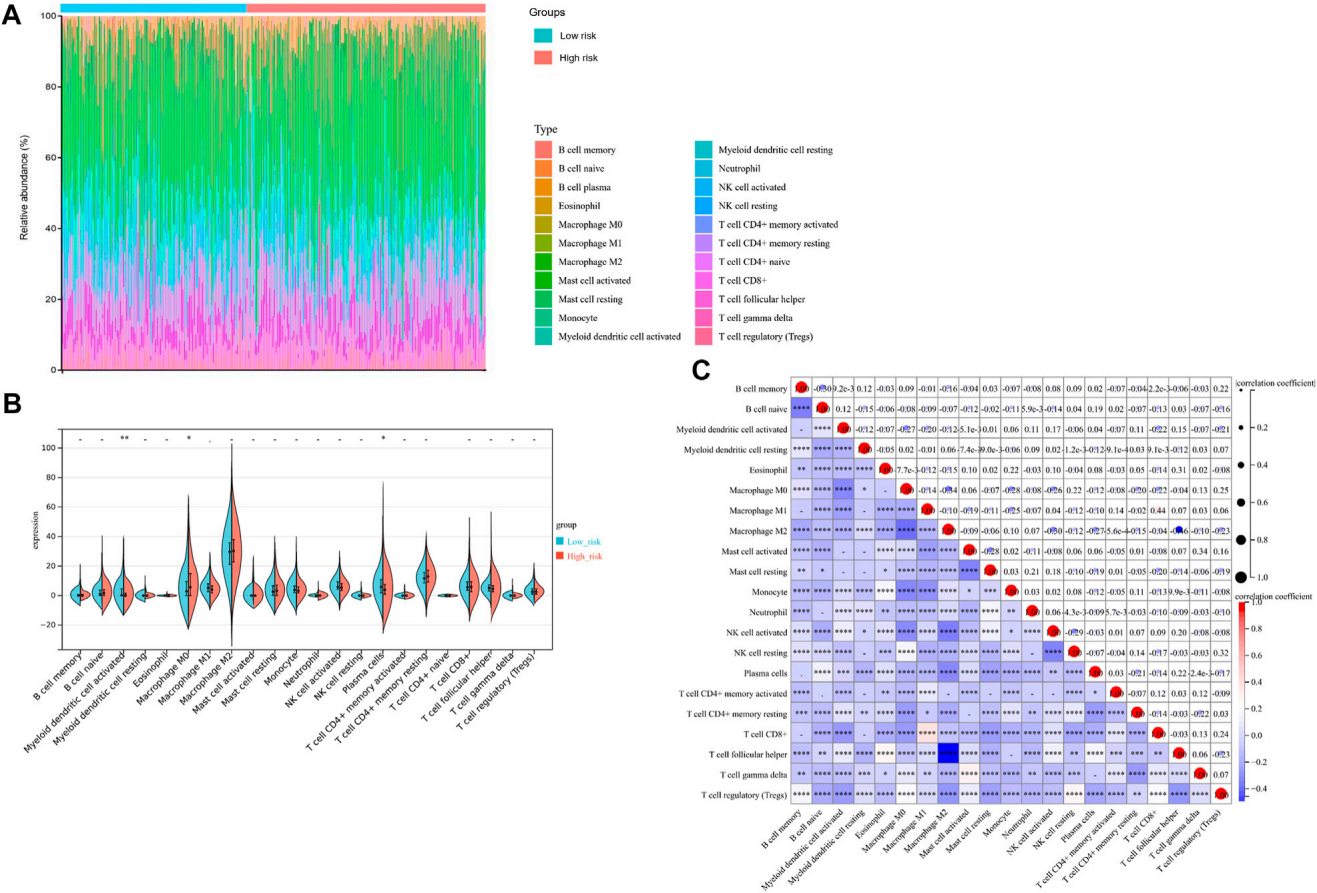
Immunity Analysis for the 3-gene Signature Risk Score and Tumor Immune Microenvironment. **(A)** The Boxplot diagram showed the composition of the 22 immune cells infiltrating in patients from low-risk and high-risk groups, based on the CIBERSORT analysis. **(B)** The Violin plot showed the difference of 22 immune cells infiltration correlations between two groups stratified by the 3-gene Signature. **(C)** The correlation matrix for the proportion of immune cells in OV patients. *p-value < .05; **p-value < .01; ***p-value < .001; ****p-value < .0001.

Additionally, we evaluated the landscape of immune infiltration among OV patients through the CIBERSORT deconvolution algorithm in order to determine whether this 3-gene signature was related to the tumor immune microenvironment. Figure 8A summarized the composition of the 22 immune cells infiltrating in OV patients from both low-risk and high-risk groups. Based on the CIBERSORT analysis, 3 out of the 22 immune cell proportions, including activated Myeloid dendritic cells (DCs), plasma cells, and M0 macrophages, were significantly different between the two risk groups. As shown in Figure 8B, activated DCs and plasma cells were downregulated in the high-risk group compared to the low-risk group, while M0 macrophages were upregulated. Correlation analysis between the 22 immune cells implied that Macrophage M1 and CD8+ T cells had the highest positive relationship, with a correlation coefficient of .44 (*p*-value < .0001) (Figure 8C). Except for the intense negative correlation between all kinds of activated cells and resting cells, Macrophage M2 and Follicular helper T cells had the strongest negative relationship, with a correlation coefficient of .46 (*p*-value < .0001).

### 3.7 Assessment of response to immunotherapy and chemotherapy in OV patients

**FIGURE 9:**
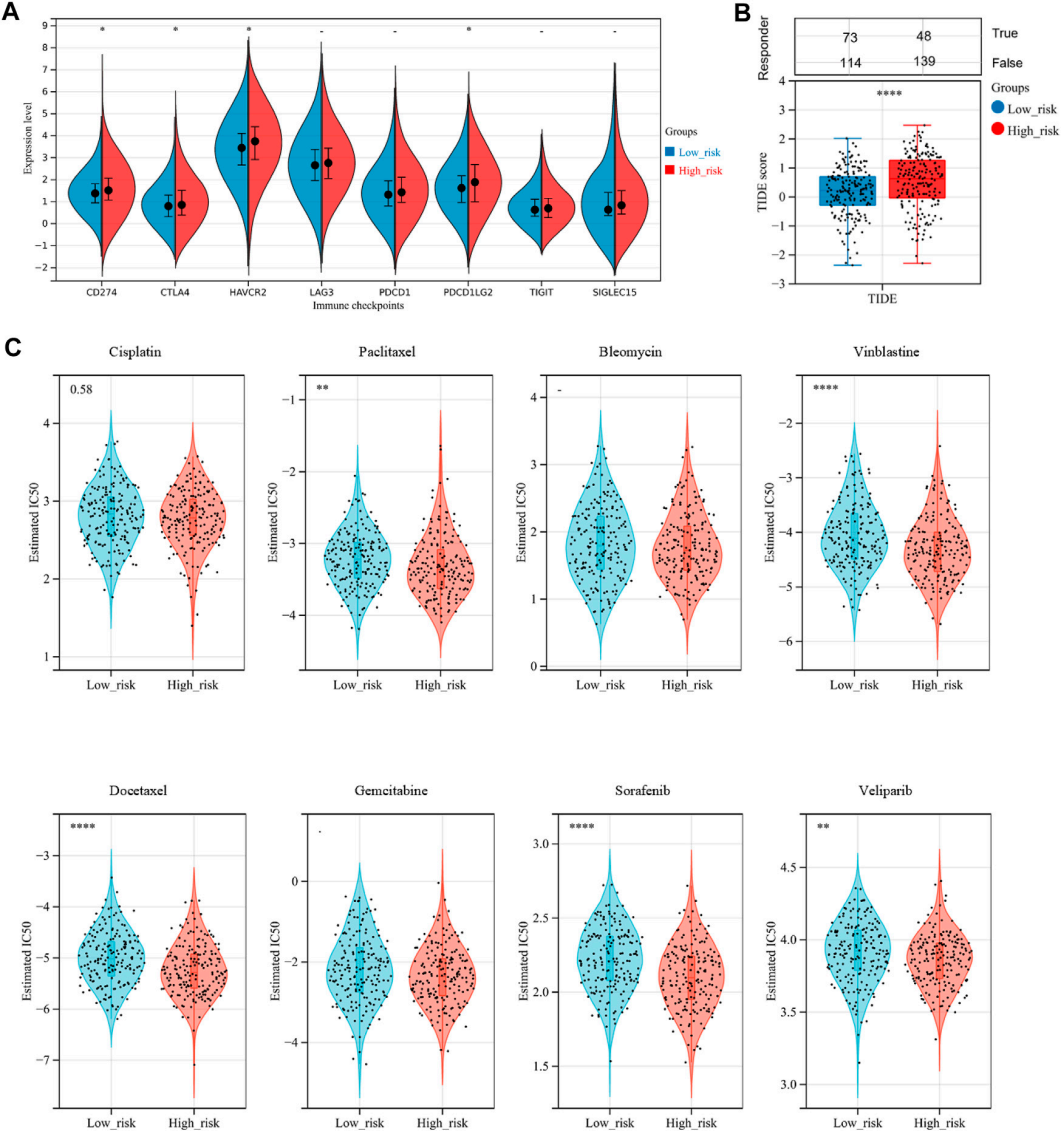
Assessment of sensitivity to immunotherapy and chemotherapy among OV patients. **(A)** The distribution of immune checkpoints gene expression between the low-risk and high-risk groups. **(B)** Immunotherapy response prediction for OV patients between the low-risk and high-risk groups, based on the Tumor Immune Dysfunction and Exclusion (TIDE) analysis. **(C)** The distribution of the estimated IC50 values for eight typical chemotherapies, including Cisplatin, Paclitaxel, Bleomycin, Vinblastine, Docetaxel, Gemcitabine, Sorafenib, and Veliparib in the Genomics of Drug Sensitivity in Cancer (GDSC) database for low-risk and high-risk groups. NS, not significant; *p-value < .05; **p-value < .01; ***p-value < .001; ****p-value < .0001.

Nowadays, mountains of evidence support the clinical implications of immune checkpoint molecules in the realm of immunotherapy for OV patients. Accordingly, we evaluated the relationships between the risk score and expressions of immune checkpoint molecules. The results implied that CD274, CTLA4, HAVCR2, and PDCD1LG2 were significantly higher in the high-risk group than in the low-risk group (Figure 9A, *p*-value < .05), suggesting that OV patients in the high-risk group could be more likely to benefit from immunotherapies related to these critical immune checkpoints. Stepwise, we applied the Tumor Immune Dysfunction and Exclusion (TIDE) algorithm to predict clinical response toward immune checkpoint blockade (ICB) in two risk groups. Interestingly, we found that OV patients in the high-risk group had greater TIDE score, which represents poorer efficacy of ICB and shorter survival after the ICB treatment (Figure 9B, *p*-value = .0001).

To assess chemotherapy sensitivity between the two risk groups, we estimated the half-maximal inhibitory concentration (IC50) of eight commonly used OV chemotherapy agents by ridge regression based on the Genomics of Drug Sensitivity in Cancer (GDSC) database (Figure 9C). Our data showed that the estimated IC50 levels of Paclitaxel, Vinblastine, Docetaxel, Sorafenib, and Veliparib in the low-risk group were significantly higher than that in the high-risk group, indicating that the OC patients in the high-risk group were more sensitive to these drugs. However, there was no significant difference in sensitivity to Cisplatin, Bleomycin, and Gemcitabine between the two risk groups.

## 4 Discussion

OV is one of the most lethal gynecological malignancies worldwide, by virtue of its inefficient detection methods and high recurrence rate (Lheureux et al., 2019). Hence, identifying reliable prognostic signatures is of great urgency. Recently, emerging research reported that ferroptosis, a newly discovered type of non-programmed cell death marked by iron accumulation and lipid peroxidation, was closely associated with various physiological and pathological processes, including cancer development (Li et al., 2021; Chen et al., 2022). As for OV, the possible connection between ferroptosis and cancer was mainly bridged through three “musketeers”: the FSP1-CoQ10 protection pathway (Doll et al., 2019), GPX4-GSH protection pathway (Zhang et al., 2019), and GCH1-BH4 protection pathway (Wei et al., 2020). In this regard, here we identified a ferroptosis-related 3-gene signature and further evaluated the prognosis value, immune microenvironment features, and treatment response of the ferroptosis patterns.

In 2021, Yang and colleagues reported a prognostic model of 9 FRGs (namely ACSL3, ALOX12, LPCAT3, PTGS2, CRYAB, HSBP1, SLC7A11, SLC1A5, and ZEB1) identified by the COX regression analysis. However, only 60 FRGs were included for signature identification, and immune analysis was not involved in the study (Yang et al., 2021a). In another previous investigation, Wang and colleagues constructed and validated a prognostic model consisted of 15 FRGs and 2 ESTIMATE scores to predict OV prognosis, though with limited 5-year ROC-AUC of .54, .61, and .54 in various cohorts (Wang et al., 2021). Up till now, no unified ferroptosis-related prognostic model has been applied to clinical practice. In the present study, in order to identify more precise ferroptosis-related mRNAs of OV patients, we downloaded gene-centric data from the Genecards database (https://www.genecards.org/), one of the largest integrative databases that could provide comprehensive information of all annotated and predicted human genes, which are integrated from ∼150 web sources. Then, we chosen a total of 182 FRGs with Relevance score ≥ 1 at this database and identified 63 potential ferroptosis-related genes from the 6,406 DEGs between the TCGA-OV and GTEx cohorts. Through the LASSO-penalized Cox analysis, we distinguished a 3-gene signature (SLC7A11, ZFP36, and TTBK2) with satisfactory prognostic value in both TCGA (*p*-value < .0001) and ICGC cohort (*p*-value = .0064). Combined with other clinical features, we also provided a promising quantitative measurement of the FRGs-related nomogram to predict the OV prognosis. To our knowledge, our study is initial to identify the 3-gene ferroptosis-related signature for OV, which could improve prognosis prediction and guide clinical decision-making. However, considering that most traditional gene-focused methods had limited clinical utility to predict individual outcomes, researchers developed methods based on the concept of network markers to provide more meaningful predictive information. For instance, Song et al. (2015) developed a systems approach to evaluate associations among gene expression patterns, potential for clinical metastases, and representative PPIs, which could uncover novel survival-related subnetwork signature in breast tumor. These networks might also form the basis of highly-accurate prognostic classification models for OV patients as well, which should be validated in future studies.

Among the 3 FGRs identified, only SLC7A11 has been reported in with function in OV progression. Hong and colleagues reported that SLC7A11, a signature protein of ferroptosis, could mediate cysteine uptake and promote glutathione (GSH) production by providing its precursor cysteine. Meanwhile, PARP inhibitors could promote ferroptosis in OV by suppressing the SLC7A11 expression (Hong et al., 2021). Another study claimed that lidocaine promoted ferroptosis in OV cells by regulating the miR-382-5p/SLC7A11 axis (Sun et al., 2021). ZFP36, as an RNA-binding protein, is a prominent inflammatory regulator linked to cancer and the autoimmunity process (Moore et al., 2018). A study of breast cancer demonstrated that ZFP36 could inhibit c-Jun expression, which resulted in the increase of Wee1 expression and prevented cell cycle progression from the S into the G2 phase (Xu et al., 2015). TTBK2, as another signature, is a key serine/threonine-protein kinase in the CK1 superfamily. Previous studies indicated that TTBK2 could phosphorylate tau and tubulin, which were implicated in tumor progression (Liao et al., 2015). In kidney carcinoma and melanoma, TTBK2 expression is associated with resistance to Sunitinib and cancer cell migration. However, the underlying mechanism of ZFP36 and TTBK2 is still unknown in OV, which deserves further exploration.

In addition, we evaluated the landscape of immune infiltration among OV patients. Three of the 22 immune cells, including activated DCs, plasma cells, and M0 macrophages, were differently infiltrated between the two risk groups. The proportion of activated DCs and plasma cells were downregulated in the high-risk group. Preclinical models demonstrated that activated DCs are required for the initiation of effective T cell responses, T cell recruitment into tumor tissue, and maintenance of effector memory T cell function, thus playing a vital role in immune regulatory responses and OV progression (Sabado et al., 2017; Lee and Radford, 2019). A recent study claimed that tumor-infiltrating plasma cells are significantly related to the CD8 (+) tumor-infiltrating lymphocytes, tertiary lymphoid structures, and superior prognosis of OV patients (Kroeger et al., 2016), which is consistent with our findings. A previous study by Zhang and colleagues demonstrated that OV cells could stimulate M0 macrophages to differentiate into M2 macrophages in the TME by activating the ERK signaling pathway, which finally resulted in tumor proliferation and migration (Zhang et al., 2020). Surprisingly, we found that only M0 macrophages were significantly upregulated in the high-risk group with poor prognosis. This finding needs further validation and exploration for the mechanism.

To date, therapeutic options for OV remain limited, with a high recurrence rate and chemoresistance (Webb and Jordan, 2017). Emerging evidence supported that ferroptosis was closely correlated with immunotherapy, one of the next frontiers in cancer (Lang et al., 2019; Jiang et al., 2020). Wang and colleagues reported that immunotherapy could enhance the effector function of CD8 (+) T cells and sensitize the tumor cells to treatment by regulating the ferroptosis process (Wang et al., 2019). Hence, we explored the differences in response to chemotherapy and ICB therapy between the two risk groups stratified by the 3-gene signature. The result revealed that the high-risk group had poorer efficacy in ICB treatment, while being more sensitive to chemotherapy, including Paclitaxel, Vinblastine, Docetaxel, Sorafenib, and Veliparib. Besides, high-risk OV patients could be more likely to benefit from immunotherapies related to the immune checkpoint molecules, including CD274, CTLA4, HAVCR2, and PD-L1. As we know, some PD-L1-positive OV patients respond poorly to PD-1/PD-L1 treatment in clinical practice (Yang et al., 2020). Accordingly, the action of immune checkpoint inhibitors could be more complicated than simply targeting immune checkpoint. Our findings on the relationship between the 3-FRGs signature and immune checkpoint response might provide some hints.

There are still some limitations of the study. Firstly, the number of samples in the TCGA-OV cohort is still limited. Hence more independent large-scale datasets are needed to verify the signature. Secondly, the underlying mechanism of the 3 identified FGRs, especially ZFP36 and TTBK2, in OV progression is still largely unknown, which needs further investigation. Last but not the least, we analyzed the RNA-sequencing expression profiles and corresponding clinical data of the TCGA-OV cohort, which was consisted of patients with ovarian serous cystadenocarcinoma. However, OV is a highly heterogeneous carcinoma with various histological subtypes, which have unique genetic, molecular, and immune profiles. Further researches were still be needed to validated our findings among each individual histological subtype.

Recently, the rapid advance of single-cell technologies leads to the growth of single-cell multi-omics data. For instance, Song and colleagues developed the Single-cell Multi-omics Gene co-Regulatory algorithm (SMGR), a novel method to detect target genes and coherent functional regulatory signals based on the single-cell assay for transposase-accessible chromatin using sequencing (scATAC-seq) and the joint single-cell RNA-sequencing (scRNA-seq) data (Song et al., 2022). Thus, in the future, with the accurate and reliable integrative analysis of single-cell multi-omics data, we might further uncover the intrinsic molecular underpinnings and underlying mechanisms of the OV prognostic signature.

## 5 Conclusion

In summary, we identified and validated a novel ferroptosis-related 3-gene signature (SLC7A11, ZFP36, and TTBK2) as an independent indicator for predicting the prognosis and treatment response of OV patients. The immune analysis supported the relationship between the ferroptosis patterns and specific immune cell population infiltration and hinted at the potential of immunotherapy in specific OV populations. Interestingly, the comprehensive analysis revealed that the signature also interacted with several vital signal pathways, such as the m6A modification, though the underlying mechanisms remain unknown so far. Thus, our findings provided new insight into the ferroptosis patterns and immune infiltration in OV, and assisted personalized treatment decision-making through a promising prediction signature for both prognosis and therapy responses.

## Data Availability

The original contributions presented in the study are included in the article/Supplementary Material, further inquiries can be directed to the corresponding author.
